# Editorial: Gut virome and human health

**DOI:** 10.3389/fcimb.2022.1043256

**Published:** 2022-10-10

**Authors:** Yuan Gao, Michael B. Sohn, Jinfeng Wang

**Affiliations:** ^1^ Beijing Institute of Genomics, Chinese Academy of Sciences/China National Center for Bioinformation, Beijing, China; ^2^ Department of Biostatistics and Computational Biology, University of Rochester, Rochester, NY, United States; ^3^ College of Food Science & Nutritional Engineering, China Agricultural University, Beijing, China

**Keywords:** intestinal tract, eukaryotic virus, phage, metagenomics, disease, host immunity

The viral community, consisting of eukaryotic viruses and phages, is the population of the most numerous microorganisms in human gut. These eukaryotic viruses and phages, along with other microbes such as bacteria and fungi, form a complex and dynamic ecosystem that influences the health of a host. Due to the limitations of experimental and analytical techniques, previous studies on gut viruses were not as widespread as those on bacterial communities. Recently, virome analysis has allowed us to have a better understanding of the viral community (Ogilvie and Jones, 2015), and knowledge about the relationship between the gut virome and human health is rapidly accumulating. For example, it has been clearly shown that phages can directly stimulate the host immune system like eukaryotic viruses and influence the physiological state of the human body besides their widely known ability to prey on bacteria and shape the intestinal ecosystem (Sausset et al., 2020). To add new insights to human virome, we organized the Research Topic: *Gut Virome and Human Health*.

This Research Topic focuses on the gut viral community associated with human physiological and pathological states, and aims to collect virome data resources, identify human commensal microorganisms more broadly, discover the interactions between gut viruses and other microbes, and explore the regulatory role of the human gut virome on intestinal microecology. From this collective effort, we hope to reveal the alterations of the human gut virome in various disease and their threats to host physiology and health, with an aim to investigate the pathogenicity of gut viruses, their response or indication to metabolic and physiological changes, their feedback to the host, and their impact on therapeutic efficacy. The articles included in this Research Topic may contribute to a better understanding of the important role that gut viruses play in human health.

First of all, the most frequently mentioned aspect of the gut virome is undoubtedly the microecological regulatory capacity of phages. Phages play a prey-predator relationship with their bacterial hosts and influence the growth of non-host bacteria through complex interactions such as symbiosis or competition, thus carving out the configuration of gut microbiota. By analyzing the gut microbiome data of over 10,000 newborns and infants from 17 countries worldwide, we suggested that phages play a significant community-shaping role in the formation of gut microbiota in early life (Xiao et al., 2021). In this Research Topic, Zuppi et al. showed a picture of the ecological network of the gut in which phages are involved, reviewing phage interactions with bacteria, other phages, host immune cells, and the immune system. Readers will see the role of phages as regulators of the intestinal ecosystem and recognize the importance of phages in maintaining the dynamic balance of the gut microbiota.

Second, the cascading regulatory effects of gut viruses on the ecosystem and host immunity will have a dramatic impact on the digestive tract and human health status. Several recent studies have reported the association between gut viruses and clinical diseases, such as inflammatory bowel disease (Norman et al., 2015), gestational diabetes (Wang et al., 2018), and cancer (Nakatsu et al., 2018). The relevance of the gut virome to disease, i.e., its potential benefits or risks to human health, has attracted increasing attention. A mini review by Spencer et al. in the Research Topic compiled a series of results on the role of the virome in gastrointestinal health and disease. They summarized the pattern of changes in healthy human intestinal phage and eukaryotic virus from infancy to old age, elaborated on the role of the virulome in a variety of intestinal diseases, and showed that the virome stimulates immunogenicity and imparts immunity to the host. They also discussed the positive effects of phage therapy on various diseases as well as how the virome stimulates host immunogenicity.

Besides the above two review articles that summarize the inextricable link between virome and digestive diseases, several new insights are also included in this Research Topic. Colorectal cancer (CRC) has been of great interest among digestive tract diseases. An interesting research paper contributed by Zuo et al. showed significant depletion of Herelleviridae in CRC patients compared to healthy individuals from analyzing metagenomic data from multiple studies in multiple countries. They also constructed a machine learning model for prognosis of CRC based on viral signals. Another interesting paper led by Li et al. analyzed the gut virome of patients with irritable bowel syndrome (IBS) and compared it with those with type 2 diabetes, Crohn’s disease, CRC, and liver cirrhosis. They explored the synergistic changes of gut viruses with bacteria and metabolites, and built a database for robustness of ecological networks, modular connectivity and functional enrichment, which provides new insights into possible positive effects of viruses on intestinal health. The results of these two research papers may be attractive to practitioners who are developing medical or diagnostic products.

This Research Topic will help us to better understand the characteristics of the gut virome and unravel some mysteries of its role in the intestinal microecosystem. However, there are still some important elements that have not been covered: 1) virome methodology is still challenging, especially a large gap in computational methods for virome; 2) studies in this Research Topic have tried to use virome signatures as markers for disease prediction, and whether or not they perform better than bacterial or traditional markers needs further evaluation; 3) Phage therapies are extremely promising but have been subject to many questions due to their safety and efficacy, which limits their clinical use and commercialization and warrants more exploration in the future. It is reassuring that the discovery and exploration of the role of the gut virome in human health and disease should be continuing.

## Author contributions

All authors listed have made a substantial, direct, and intellectual contribution to the work and approved it for publication.

## Funding

It is supported by the National Natural Science Foundation of China (31870107 and 32070122) and Chinese Universities Scientific Fund (2022RC027).

## Acknowledgments

The authors are grateful to all the authors, reviewers and editors who have participated in this Research Topic for their excellent contributions.

## Conflict of interest

The authors declare that the research was conducted in the absence of any commercial or financial relationships that could be construed as a potential conflict of interest.

## Publisher’s note

All claims expressed in this article are solely those of the authors and do not necessarily represent those of their affiliated organizations, or those of the publisher, the editors and the reviewers. Any product that may be evaluated in this article, or claim that may be made by its manufacturer, is not guaranteed or endorsed by the publisher.
